# The role of routine imaging in identifying endoluminal colorectal pathology, a United Kingdom clinical experience

**DOI:** 10.1007/s00261-025-05255-6

**Published:** 2025-12-22

**Authors:** Narendranath Govindarajah, Daniel Livingstone, Robert Mitchell, Keith Farrell-Dillon, Edward Antram, Sharmin Malekout, Nigel Beharry, Kunal Patel, Nirav Patel, Kesavan Kandiah, Anita Wale

**Affiliations:** 1 Department of Radiology, St George’s Hospital, London, UK; 2 Molecular and Clinical Sciences Research Institute, City St George’s, University of London, London, UK

**Keywords:** Colorectal neoplasms, X-ray computed, Radiology, Tomography, X-ray computed + colonoscopy or virtual colonoscopy

## Abstract

**Purpose:**

Colorectal cancer (CRC) is a leading cause of cancer-related death, and timely and accurate recognition of endoluminal pathology is crucial. While CT colonography (CTC) is validated for luminal assessment with a sensitivity concordant to endoscopy, most radiology referrals to the tumor board are based on unprepared CT abdominal studies without bowel preparation or fecal tagging. The diagnostic yield of these routine unprepared CT scans and the influence of radiologist subspecialty, remain uncertain. This study evaluated the positive predictive value of CTC and unprepared CT for suspected endoluminal pathology and examined the impact of gastrointestinal (GI) subspeciality reporting.

**Methods:**

We reviewed colorectal tumor board outcomes from 2022 at St George’s Hospital, London, United Kingdom. Patients referred to the tumor board by radiology were identified and analyzed through patient records. Radiological and endoscopic concordance was assessed using composite endpoints.

**Results:**

Of the 106 radiology-initiated referrals to the tumor board in 2022, 61 (58%) were for suspected endoluminal pathology. Overall positive predictive value (PPV) was 79% (42 true positives and 11 false positives). The PPV was 91% for CTC and 70% for unprepared CT. GI subspecialist reporters identified 44% more endoluminal lesions on unprepared CT than non-specialist reports (*p* < 0.0001), but without a significant difference in PPV (67% vs. 78%, *p* = 0.543). No significant difference in colorectal cancer detection was observed between CTC and unprepared CT (*p* = 0.8).

**Conclusion:**

Unprepared CT demonstrates a good PPV (70%) for detecting endoluminal pathology, with over half of identified lesions being malignant. Although PPV is comparable between GI and non-GI radiologists, GI subspecialists refer significantly more cases for further evaluation, emphasizing the importance of subspeciality expertise. Radiologists should confidently raise suspicion of endoluminal pathology to ensure timely referral for direct visualization and tumor board discussion.

## Background

154,270 cases of colorectal cancer (CRC) are diagnosed in the US annually [1]. In the UK more than 42,000 cases of colorectal cancer (CRC) are diagnosed annually. It remains the second most common cause of cancer-related mortality, with an overall incidence increase of 5% over the last decade [2]. In the UK patients are diagnosed through several pathways: the majority (55%) via referral from primary care, 10% through screening via the national Bowel Cancer Screening Program (BCSP), approximately 18% present as an emergency, and 17% have unclear referral sources [3]. CRC represents a significant health burden, both in terms of incidence and the resources devoted to screening. This burden is growing, driven by rising incidence among younger populations, heightening clinical concern regarding early cancers.

The UK was the first country to establish a colorectal multidisciplinary team (tumor board) in 1995, following recommendations from the Calman-Hine report aimed at standardizing CRC care nationally [4]. The tumor board process has contributed to improved survival rates, partly by increasing the proportion of patients with advanced CRC who receive post-operative chemotherapy after individualized case reviews [5]. Traditionally, tumor board discussions focus on patients with confirmed cancer. However, at our hospital, patients flagged with suspicious imaging findings are discussed in the tumor board until CRC diagnosis is either confirmed or excluded, as part of a fail-safe system [6]. Currently, there is no defined national pathway in the UK for direct referral to the colorectal tumor board from Radiology.

Colonoscopy remains the gold standard for direct visualization of the large bowel but carries inherent risks and is a limited resource. Alternative investigative strategies have evolved, including CT colonography (CTC) and fecal immunochemical testing (FIT) for detecting microscopic blood in stool. The SIGGAR multi-center randomized controlled [7] trial demonstrated that CTC has a sensitivity of 96% for detecting CRC and polyps > 6 mm compared to colonoscopy, though it is primarily used when endoluminal pathology is suspected or being screened for [7]. Most patients undergoing CT without suspicion of CRC receive CT abdomen and pelvis studies with or without intravenous contrast (unprepared CT). The sensitivity of unprepared CT for CRC diagnosis varies widely (75–100%), depending on cohort, sample size, and diagnostic criteria [8], with evidence to support that unprepared CT was only moderately effective in diagnosing invasive cancers [9] and limited data available for benign or premalignant disease. A recent retrospective blinded study by Wahlig et al. [10] reported sensitivities of 64–77% and specificities of 83–86% for CRC detection using unprepared CT. Wahlig et al. [10] analyzed these metrics in a retrospective case-controlled study with repeat imaging and known pathology.

In this study, we aimed to describe our real-world experience investigating CT-identified endoluminal pathology.

## Methods

This study was reviewed by the Research Ethics and Integrity Officer at the Joint Research and Enterprise Services, St George’s School of Health and Medical Sciences.

City St George’s, University of London (UK) and was deemed to meet the local requirements for service evaluation (Registration Number: AUDI003923). The requirements for ethical approval and informed consent were waived in adherence to the Hospital’s privacy notice and the NHS Data Opt-out.

### Patients

**Fig. 1:**
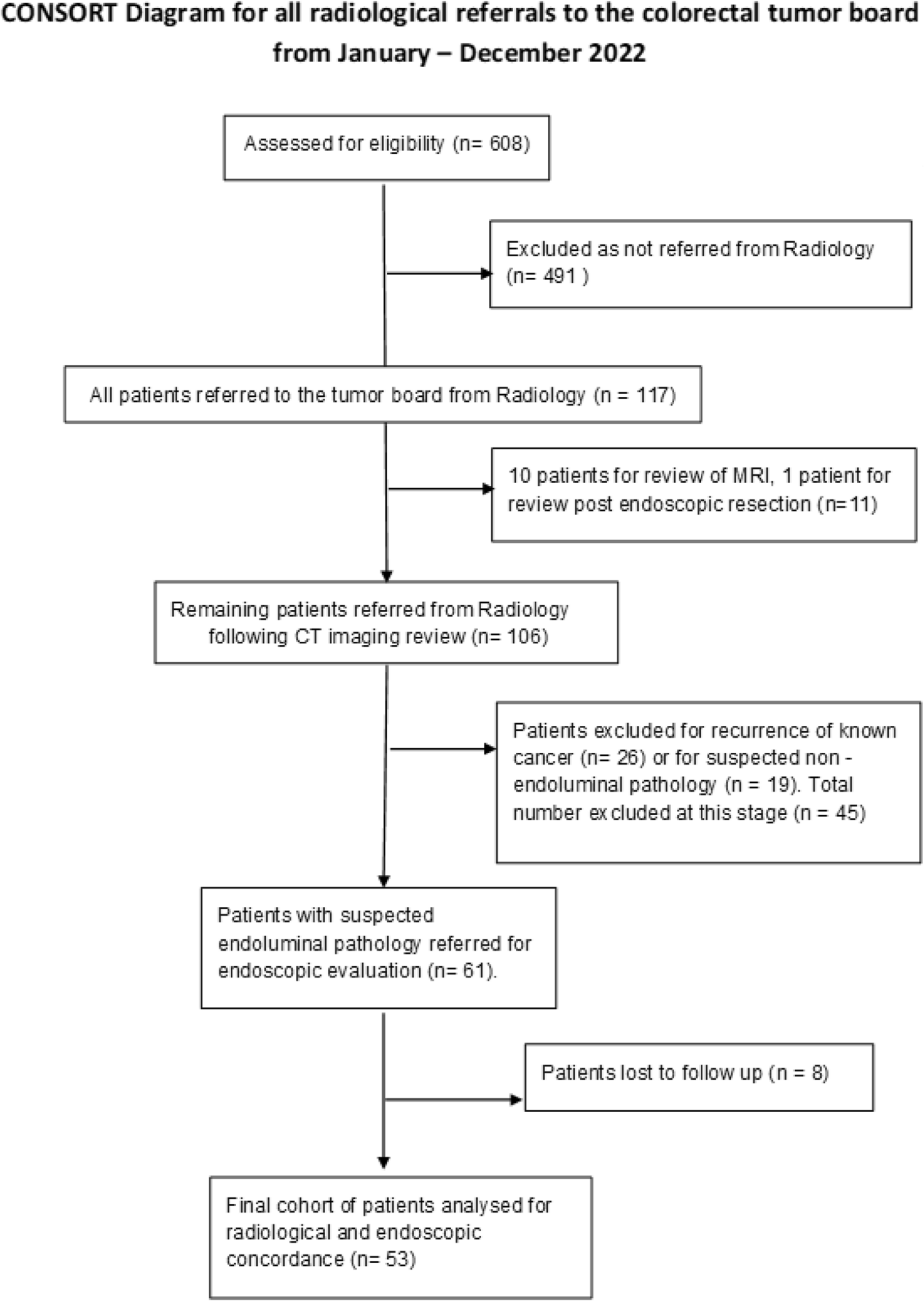
CONSORT diagram to illustrate the patient cohort referred for tumor board discussion from January–December 2022 with flow of exclusions and final analyzed cohort

St George’s Hospital is a regional colorectal cancer screening unit. Colorectal tumor board outcomes from 7th of January 2022–30th December 2022 at St George’s Hospital, London, U.K. (SGH) were reviewed from a prospectively maintained database. Six hundred and eight patients (*n* = 608) patients were discussed at the colorectal tumor board over this time. The sample was filtered to include only those whose referral for discussion in the lower-GI tumor board was “radiology-initiated” via our local fail-safe alerting pathology where imaging which may represent malignancy or progression of known malignancy are referred for tumor board discussion therefore, *n* = 117 (19%) of all the tumor board discussions were “radiology-initiated”. Eleven patients (*n* = 11) were excluded as they were referred for review of specific MRI findings (*n* = 10) or documentation reasons only (*n* = 1). Forty-five patients (*n* = 45) were then excluded as they were referred for discussion of; (A) recurrence of known cancer (*n* = 26) or (B) for suspected non-endoluminal pathology (*n* = 19). Sixty-one (*n* = 61) patients were referred for endoscopic evaluation following the imaging findings, following this, 8 patients were excluded as lost to follow up leaving a final cohort of 53 patients analyzed for endoscopic vs. radiological concordance (*n* = 53). No patient had two radiology-initiated discussions in the tumor board over the course of the study period. Some patients were discussed more than once but there were only 608 individual patients discussed over the course of the study (Fig. 1).

### Imaging technique

Unprepared CT (CT abdominal pelvis (CTAP)) scans were acquired on two scanners. These were both 128 slice General Electric (GE) machines. Contrast was administered using weight-based volume calculation of Omnipaque 300 (normally 90 ml) injected at 3 ml/s and a delay of 65–70 s for image acquisition in the portal venous phase with coverage from diaphragm to pubic symphysis.

For CT colonography (CTC) all studies were performed on a Siemens Somatom X.cite Dual Energy machine 128-slice. Bowel preparation was administered in accordance to departmental guidelines using Gastografin fecal tagging and catharsis with low residue diet. Contrast enhanced imaging was performed using weight-based contrast for patients presenting with symptoms (not screening).

### Imaging, follow up and outcomes

Original imaging reports were reviewed and recorded for the presence of suspected endoluminal pathology. Suspected endoluminal pathology was deemed to be mural thickening, focal lesion or evidence of endoluminal narrowing on imaging. The subspecialist training of the reporting radiologist was recorded. If a scan was reported by multiple radiologists (for example if a registrar provided an initial report which was then authorized by a consultant) the subspecialist training of the most senior radiologist was recorded. 14/117 (12%) of examinations were not reported by radiologists in our hospital but by external reporting companies, these were recorded separately but scored as non-GI radiologists for the purposes of analysis unless we were able to find documentation of the reporters GI subspeciality training status (two cases). All CT colonography examinations were assumed to be reported by a GI subspeciality trained radiologist. Endoscopic records and the electronic patient records were interrogated for the performance of colonic endoscopy (flexible sigmoidoscopy or colonoscopy) and the endoscopic findings.

The imaging dataset was not re-reviewed according to our aim of representing a “real-world” experience, bias was therefore reduced.

Patients were followed up until tumor board discussion determined the nature of the imaging findings which resulted in the “radiology-initiated” discussion. Outcome follow up was completed in March 2024, thus at least 18 months from the scan.

### Outcomes/endpoint data

If the patient had suspected endoluminal pathology and endoscopic evaluation was performed, the endoscopic findings were regarded as the gold standard. If, however, the patient was unable to undergo endoscopic evaluation the clinical notes were interrogated to determine the nature of the finding from any subsequent surgery or imaging examinations. These combined approaches formed the composite endpoint to determine the nature of any endoluminal pathology. Any patients lost to follow-up or where the nature of the endoluminal pathology could not be identified are highlighted as missing values.

Imaging and endoscopic findings/composite endpoint were compared for concordance, assuming the imaging was the investigative test, and the endoscopy/composite endpoint was the gold standard. True positive CT findings were those where an abnormality was demonstrated at the same site either on endoscopy or follow-up and false positive CT findings were those where the colon was normal at this site either on endoscopy or on follow-up. CT colonography findings are included as a comparator study to unprepared CT as they are the validated gold standard imaging modality for the investigation of endoluminal pathology.

### Statistical analysis

Formal sample size calculations were not performed as this was a hypothesis-generating service evaluation. The primary outcome was the true positive rate of suspected endoluminal findings on CT, compared to endoscopy as the gold standard. Descriptive statistics were used with MS Excel (v 2308) and Medcalc software. Differences in proportions were assessed by Chi squared or Fishers exact test dependent on sample size and a two-tailed z test was performed as appropriate. Where relevant 95% confidence intervals are reported. *P* < 0.05 is considered statistically significant. Continuous variables were reported as recorded [11].

## Results

### Patient demographics

A final cohort of 61 patients were included, each of whom were referred for discussion of CT findings of suspected endoluminal pathology directly from the Radiology Department. 38/61 (62%) were male, with a mean age of 72 years (range 40–92 years, SD 13.3 years). The cohorts of patients imaged with CT colonography and unprepared CT were matched in terms of their key demographics (Table 1).

**Table 1:** Demographics

Characteristic		Unprepared CT (*n* = 34)	CT colonography (*n* = 27)	*P* value
Age	Mean (SD)	70 (15.2)	74 (10.3)	0.2468*
	Range	40–92	47–89	
Sex	Male	20 (59%)	18 (67%)	0.6**
	Female	14 (41%)	9 (33%)	
Sub-specialism of the reporter	GI	22 (65%)	27 (100%)	
	Non-GI	12 (35%)		

*P value determined with the Student’s T test, **P value determined with Fisher’s Exact Test

The patients had a variety of CT scans which had identified a finding for discussion in the tumor board (Table 2), the most common of which was CT thorax, abdomen and pelvis with contrast (*n* = 34) followed by CT colonography (*n* = 27).

### Endoluminal pathology on CT (in patients without history of colorectal cancer)

61/106 patients had suspected endoluminal pathology on CT imaging (58%) – 27/61 (44%) were CT colonography examinations and 34/61 (56%) were unprepared CT. 45/61 (73%) of these patients underwent an endoscopic investigation, 20/27 flagged from CT colonography and 25/34 from unprepared CT). Of the 16 who did not undergo endoscopic evaluation, the nature of the endoluminal findings could be determined from interrogation of the patients notes for 8 patients but 8 were lost to follow up. Therefore, the nature of the CT identified endoluminal pathology could be assessed in 53/61 (87%) patients.

**Table 2:** Demographic and diagnostic characteristics of patients undergoing unprepared CT or CT colonography for evaluation of endoluminal pathology

		Unprepared CT			CT colonography			Total
		Enhanced	Unenhanced	Total	Enhanced	Unenhanced	Total	
Number		32	2	34	24	3	27	61
Gender	Male	19	1	20	15	3	18	38
	Female	13	1	14	9	0	9	23
Age	Mean (SD)	70 (16)	71 (9)	70 (15)	73 (10)	79 (10)	74 (10)	72 (13)
SD	Range	40–92	65–77	39–92	47–89	68–88	47–89	40–92
Subspecialty of reporting radiologist	GI	20	2	22	24	3	27	49
	Non GI	12	0	12	0	0	0	12
Outcome								
	Missing	4	0	4	2	2	4	8
	FP	8	1	9	2	0	2	11
	TP	20	1	21	20	1	21	42
Nature of TP								
	Benign polyp	4	0	4	6	1	7	11
	Benign inflammatory	3	0	3	3	0	3	6
	Malignant	13	1	14	11	0	11	25

Table 2. summarizes the number of patients, gender distribution, age (mean, standard deviation and range), radiologist subspeciality and diagnostic outcomes (true positives (TP), false positives (FP) and missing data values) across imaging modalities. Subgroups are divided by contrast enhancement (enhanced vs. unenhanced) and reporting subspecialty (GI vs. non-GI). Nature of true positives is further classified as benign polyp, benign inflammatory or malignant lesions.

Of the 34 patients who had unprepared CT, 30 had outcomes data available. 21/30 had a true positive endoluminal abnormality, benign or malignant, (16 determined by endoscopy, 5 determined by composite endpoint) resulting in a 70% positive predictive value.

70% of all cases were reported by a GI radiologist and 30% by a non-GI or external radiologist. GI radiologists identified 14 true positive endoluminal abnormalities and 7 false positive abnormalities (PPV 67%) whereas non-GI radiologists identified 7 true positive endoluminal abnormalities and 2 false positive abnormalities (PPV 78%), z = 0.608, *p* = 0.543. Therefore, out of the 30 unprepared CT’s that were performed where outcome data was available, 40% more suspected endoluminal pathology was identified by GI radiologist reporting (95% CI 21.13–55%, *p* < 0.0001) but with no difference in positive predictive value.

CT colonography had a 91% positive predictive value for the detection of endoluminal pathology with 21/23 patients showing endoluminal pathology at the site of endoluminal disease.

### Nature of endoluminal findings

**Fig. 2:**
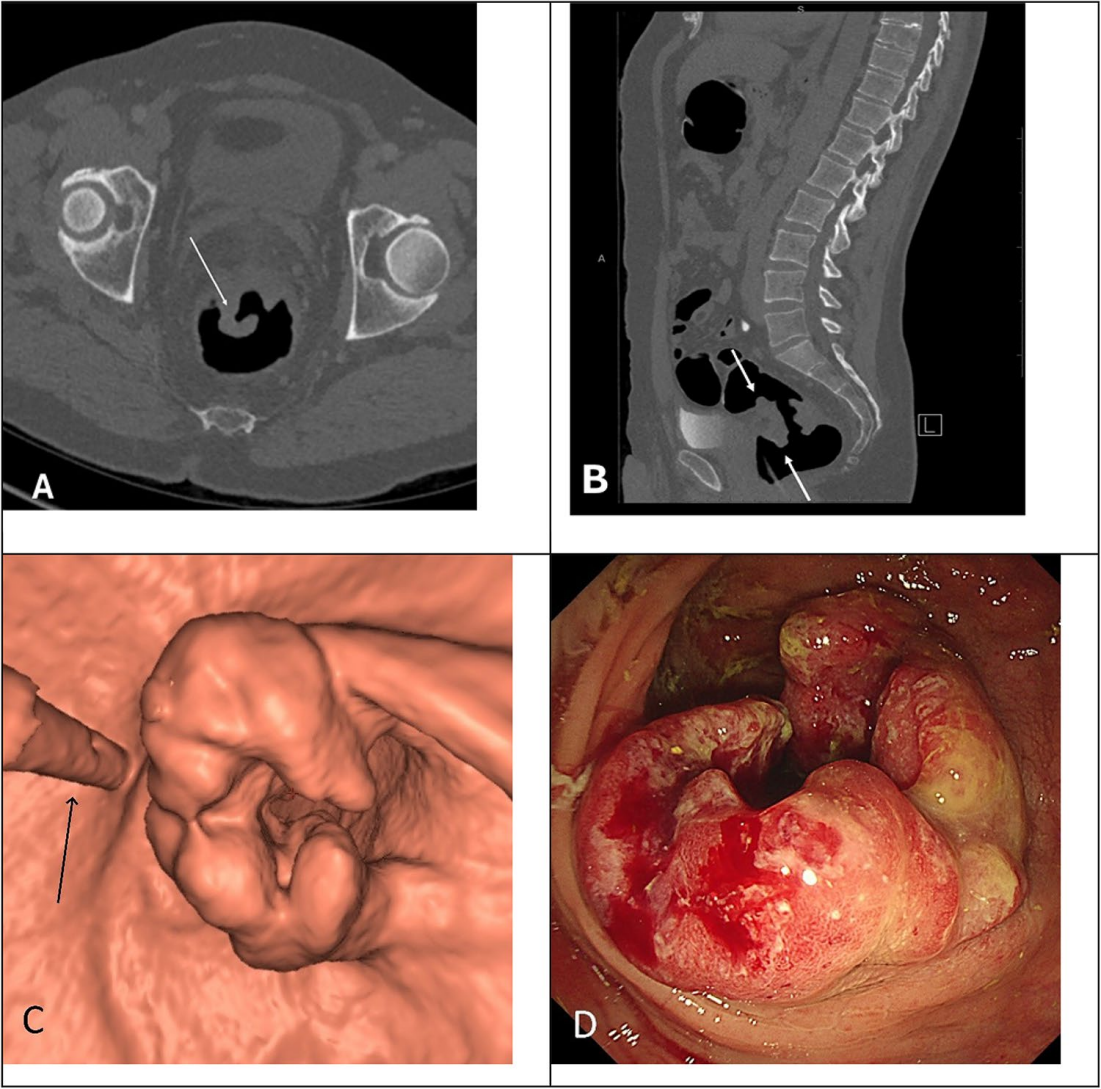
CT colonography examination and endoscopic images of a patient with a locally advanced rectal cancer (subsequently staged by MRI as T4a, N1c, EMVI positive, CRM involved, PSW negative). **A** shows supine axial view and **B** the prone sagittal view of the semi-annular lesion in the rectum (solid white arrow), note the rectal catheter (black arrow). Virtual colonoscopic image is shown in **C** (note the rectal catheter, black arrow) and the endoscopic image in **D** (note the different orientation between endoscopic and CT images). *EMVI* extramural vascular invasion, *CRM* circumferential resection margin, *PSW* pelvic side wall lymph node

**Fig. 3:**
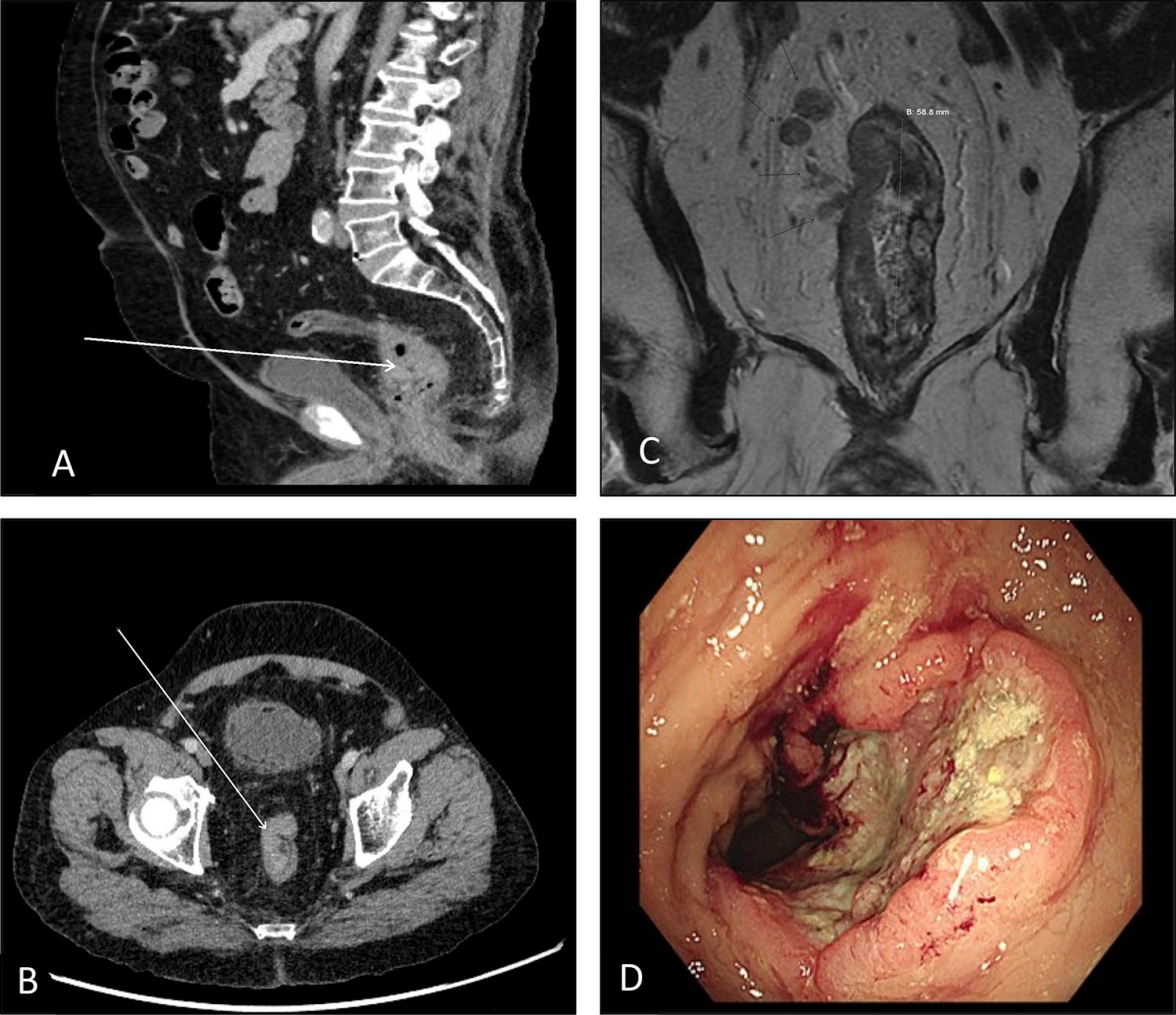
CTAP study demonstrating a mid-rectal lesion (white arrows **A** and **B**). **C** shows the subsequent T2-weighted coronal oblique small field of view MRI scan performed for local staging which demonstrates a 58 mm long tumor with further prognostic information including extramural vascular invasion (EMVI) and N1c nodal deposits (black arrows). The corresponding endoscopic image of the mid-rectal tumor is also included (**D**). This tumor was staged as T3d, N1c, EMVI positive, CRM involved, PSW negative, M0

21/42 (50%) true positive lesions proceeded to surgery, 8 of which were benign polyps (tubulovillous adenomata) and 13 were proven malignant adenocarcinoma. Table 3 describes in detail the size, nature and staging of these resected true positive endoluminal lesions. Figures 2 and 3 are selected cases of colorectal cancers which demonstrate radiological and endoscopic concordance from the selected patient cohort.

The goal of these studies is not necessarily to always identify malignancy but to identify any target lesions for colonoscopy including polyps which may be benign. Both unprepared CT and CT colonography identified more malignant than benign disease. 14/35 unprepared CT vs. 11/25 CT colonography examinations identified a malignant lesion (*p* = 0.8). There was therefore no significant difference in the diagnostic yield of unprepared CT vs. CT colonography in this sample.

### Stage of endoluminal findings identified

**Table 3:** Comparison of lesion characteristics and staging between unprepared CT and CT colonography in patients with endoluminal pathology confirmed on histology

		Endoluminal pathology present at endoscopy, surgical pathology available	
		CT non-dedicated (*n* = 10)	CT colonography (*n* = 11)
Lesion size median combined (benign & malignant) (cm)		2.8 cm	2.75 cm
Benign (*n* = 8)			
Lesion size median (range)/mm		6 mm (6–9 mm)	5.5 mm (3–24 mm)
	TVA	3	5
*Malignant (adenocarcinoma) (*n* = 13)			
Lesion size median (range)/cm		5 cm (2–5 cm)	5 cm (3–7 cm)
pT stage	X		
	1		
	2	1	
	3	4	5
	4	2	1**
pN stage	0	3	3
	1	3	3
	2	1	
ctM stage	0	6	6
	1	1	

Lesion size is presented as median values (with range) for combined benign and malignant findings. Histopathological (pT, pN) and clinical metastatic staging (ctM) are reported for malignant cases

All benign lesions were TVAs

**1 patient was down-staged, final staging was ypT4a

## Discussion

This study aimed to explore the role of radiologist-identified endoluminal pathology within the large bowel in patients without a prior history of colorectal cancer. In our cohort, 61/106 (58%) radiology-initiated referrals to the lower GI tumor board were for suspected endoluminal pathology identified on CT. CTC demonstrated a positive predictive value (PPV) of 91% for all endoluminal pathology, compared to 70% for unprepared CT. Of the true positive lesions detected, 52% were malignant on CTC compared to 67% on unprepared CT. Gastrointestinal (GI) subspecialty reporting identified 44% more endoluminal pathology on unprepared CT (95% CI 21.13–55%, *p* < 0.0001), without significant change in the PPV for this relatively small sample size.

CTC is widely regarded as the gold standard for detecting endoluminal pathology by imaging, The SIGGAR multi-center randomized controlled [7] trial demonstrated that CTC has a sensitivity of 96% for detecting CRC and polyps > 6 mm compared to colonoscopy, though it is primarily used when endoluminal pathology is suspected or being screened for [7]. Most CTC examinations performed at our institution were performed for symptomatic patients. In such cases, contrast-enhanced CTC is advised to improve the sensitivity of detecting extra-colonic pathology [12, 13] which accounts for the high proportion of contrast enhanced CTC in our cohort. Our local practice of red-flagging any imaging findings suggestive of malignancy to the tumor board likely reflects the true diagnostic yield of CTC in this setting. It is therefore notable that no significant difference found between CT colonography and unprepared CT in the detection of colorectal cancer (*p* = 0.8).

The majority of malignant lesions on unprepared CT were reported by GI subspecialists, suggesting the diagnostic performance of unprepared CT may be underestimated in general practice. Supporting this interpretation, lesions identified on unprepared CT in our study tended to be larger and more advanced than those detected on CT colonography, consistent with findings by Wahlig et al. [10].

The role of unprepared CT in detecting endoluminal pathology is less well established than that of CTC. Our findings suggest that radiologist interpretation of unprepared CT for endoluminal pathology (benign and malignant) is more accurate than previously reported. Prior studies have largely focused on sensitivity or specific patient subsets [14], and reported a lower PPV, such as the 33% reported by Colvin et al. [15] and noted that up to 20% of cancers were missed on CT before diagnostic colonoscopy [16]. The higher PPV observed in our cohort (and the consistency of our CT colonography PPV with published literature [17]) suggest that the diagnostic contribution of unprepared CT may have been underestimated.

Several factors may explain the improved PPV of unprepared CT in our study. Advances in CT acquisition protocols and post processing techniques over the past decade have likely enhanced lesion detection beyond those reported in earlier studies. A degree of referral or selection bias may also be present, as increasing awareness of colorectal cancer, particularly in younger patients, may also lower the threshold for requesting cross-sectional imaging and increase pre-test probability. Finally, institutional factors such as regular discrepancy review meetings, multidisciplinary collaboration and a strong culture of subspecialty engagement may contribute to interpretive accuracy, reflecting a broader depth of gastrointestinal radiology expertise across teams.

Our study complements Wahlig et al. [10] who demonstrated variability in radiologists’ ability to identify colorectal cancers in patients with and without known CRC. Their methodology sought to reduce bias by having radiologists review cases without prior knowledge of cancer presence. They reported individual PPVs of 0.68 and 0.67 for colorectal cancer, with combined PPVs ranging from 0.59 to 0.86, highlighting the diagnostic challenges and discrepancies often encountered in clinical practice.

In contrast, our study reflects real-world practice, and demonstrates similar PPVs between GI and non-GI radiologists. This suggests that while unprepared CT should not be used for screening, all radiologists play an important role in flagging potential endoluminal pathology. Notably, our study is the first to report outcomes of non-dedicated radiologist reporting of endoluminal pathology albeit limited by sample size, emphasizing the important role of all radiologists in identifying colorectal cancers and precursor lesions. In our data, PPV did not differ significantly between specialist and non-specialist reporters; however, GI subspecialists referred significantly more cases. These findings emphasize both the added value of subspecialty expertise and the crucial contribution of non-specialists in detecting colorectal cancer and precursor lesions.

Strengths of our study include the inclusion of both CT colonography and unprepared CT examinations referred to the lower GI tumor board, and the inclusion of non-malignant lesions. Our CTC PPV is consistent with published data, confirming the quality of GI subspecialist reporting. PPVs stated are intentionally for both malignant and benign lesions as presenting only malignant lesions undervalues the importance of detection of precursor lesions. Potential strategies to further improve diagnostic performance could include standardized reporting criteria, double-reading in equivocal cases, confirmatory CT colonography, and structured reporting templates to reduce inter-observer variability.

Our findings should reassure clinicians and radiologists that a relatively high threshold for subspecialist review and tumor board discussion is appropriate, given that a substantial proportion of cases will have genuine endoluminal pathology.

Limitations include the absence of a control cohort without suspected endoluminal disease, though the data from Wahlig et al. [10] data partly mitigates this. This single-centre design with histopathology only available for those patients who underwent resection is a further limitation, however, our center is a high-volume colorectal unit with over 600 patients reviewed annually at tumor board and approximately 100 resections performed per year. Another limitation is that only patients with abnormal CTs were included, preventing assessment of true negative and false negative rates. Future matched cohort studies could explore these metrics, but given CT’s limitations, negative predictive value (NPV) is likely poor for excluding endoluminal pathology. While identifying the specific imaging feature that trigger suspicion for endoluminal pathology was beyond the scope of this study, previous work by Wahlig et al. [9, 10] has evaluated such factors.

## Conclusion

Radiology-initiated referrals account for 19% of lower GI tumor board cases annually at our institution. While CT colonography remains the established gold standard for identifying endoluminal pathology, this study demonstrates a high PPV for radiologist-identified suspected pathology on unprepared CT studies, including both malignant and benign lesions. While the PPV is comparable between GI and non-GI radiologists, GI subspecialists refer significantly more cases for further evaluation. These findings highlight the important role of radiologist-initiated referrals in ensuring timely and appropriate colorectal cancer investigation and management.

## Data Availability

No datasets were generated or analysed during the current study.

## References

[1] National Cancer Institute. Colorectal Cancer - Cancer Stat Facts. SEER Cancer Stat Facts: Colorectal Cancer. 2022;

[2] Morgan E, Arnold M, Gini A, Lorenzoni V, Cabasag CJ, Laversanne M, et al. Global burden of colorectal cancer in 2020 and 2040: Incidence and mortality estimates from GLOBOCAN. Gut. 2023;72(2). 10.1136/gutjnl-2022-327736 36604116

[3] National Cancer Auditing Collaboration (NATCAN). NBOCA Annual Report 2019. 2019.

[4] Miles RA, Kerr DJ. The calman report on cancer services. Curr Obstet Gynaecol. 1995;5(4).

[5] MacDermid E, Hooton G, Macdonald M, Mckay G, Grose D, Mohammed N, et al. Improving patient survival with the colorectal cancer multi-disciplinary team. Colorectal Disease. 2009;11(3). 10.1111/j.1463-1318.2008.01580.x 18477019

[6] Academy of Royal Medical Colleges. Alerts and notification of imaging reports Recommendations [Internet]. 2022 [cited 2025 Mar 9]. Available from: https://www.aomrc.org.uk/wpcontent/uploads/2022/10/Alerts_notification_imaging_reports_recommendations_1022.pdf

[7] Halligan S. CT colonography for investigation of patients with symptoms potentially suggestive of colorectal cancer: A review of the UK SIGGAR trials. Vol. 86, British Journal of Radiology. 2013. 10.1259/bjr.20130137 PMC3664986 23568360

[8] Mangat S, Kozoriz MG, Bicknell S, Spielmann A. The Accuracy of Colorectal Cancer Detection by Computed Tomography in the Unprepared Large Bowel in a Community-Based Hospital. Canadian Association of Radiologists Journal. 2018;69(1). 10.1016/j.carj.2017.12.005 29458958

[9] Ozel B, Pickhardt PJ, Kim DH, Schumacher C, Bhargava N, Winter TC. Accuracy of routine nontargeted CT without colonography technique for the detection of large colorectal polyps and cancer. Dis Colon Rectum. 2010;53(6). 10.1007/DCR.0b013e3181d5de13 20485005

[10] Wahlig S, Hassan O, Behr S. Colorectal cancer detection on routine abdomen/pelvis CT. The Royal College of Radiologists Open 2, 2024, 100157.

[11] MedCalc Statistical Software Version 23.2.1. MedCalc [Internet]. 2024. Available from: https://www.medcalc.org/calc/comparison_of_proportions.php

[12] Standards of practice for computed tomography colonography (CTC). Joint guidance from the British Society of Gastrointestinal and Abdominal Radiology and The Royal College of Radiologists [Internet]. 2021. Available from: www.rcr.ac.uk

[13] Spreng A, Netzer P, Mattich J, Dinkel HP, Vock P, Hoppe H. Importance of extracolonic findings at IV contrast medium-enhanced CT colonography versus those at non-enhanced CT colonography. Eur Radiol. 2005;15(10). 10.1007/s00330-005-2798-6 15965661

[14] Myo K, Manda V, Qi LJ, Rawlings D, Leung E. Sensitivity of routine CT abdomen and pelvis for detecting colorectal cancer. International Journal of Surgery. 2016;36.

[15] Colvin H, Lukram A, Sohail I, Chung KT, Jehangir E, Berry J, et al. The performance of routine computed tomography for the detection of colorectal cancer. Ann R Coll Surg Engl. 2013;95(7). 10.1308/003588413X13629960049072 PMC5827289 24112491

[16] Klang E, Eifer M, Kopylov U, Belsky V, Raskin S, Konen E, et al. Pitfalls in diagnosing colon cancer on abdominal CT. Clin Radiol. 2017;72(10). 10.1016/j.crad.2017.06.007 28687169

[17] Horvat N, Raj A, Liu S, Matkowskyj KA, Knezevic A, Capanu M, et al. CT colonography in preoperative staging of colon cancer: Evaluation of FOxTROT inclusion criteria for neoadjuvant therapy. American Journal of Roentgenology. 2019;212(1). 10.2214/AJR.18.19928 PMC7959265 30422707

